# Therapeutic Effects of a New “Indigenous Vaccine” Developed Using Novel Native “Indian Bison Type” Genotype of *Mycobacterium avium* Subspecies *paratuberculosis* for the Control of Clinical Johne's Disease in Naturally Infected Goatherds in India

**DOI:** 10.4061/2010/351846

**Published:** 2010-02-14

**Authors:** S. V. Singh, P. K. Singh, A. V. Singh, J. S. Sohal, M. C. Sharma

**Affiliations:** Veterinary Microbiology Laboratory, Animal Health Division, Central Institute for Research on Goats, Makhdoom, Farah, Mathura 281 122, India

## Abstract

Therapeutic efficacy of an “Indigenous vaccine” has been evaluated with respect to a commercial vaccine (Gudair, Spain), for the control of clinical Johne's disease (JD) in naturally infected goatherds. Seventy-one goats (JD positive) were randomly divided into 3 groups (“Bison”, “Gudair” and “Sham-immunized”). After vaccination, goats were monitored for physical condition, morbidity, mortality, body weights, shedding of *M. paratuberculosis* (MAP) in feces, internal condition and lesions, as well as humoral and cell-mediated immune responses for 210 days. Study showed marked overall improvement in physical condition of vaccinated goats and average body weight gain was significantly higher (*P* < .05) in “Bison” group as compared to “Sham-immunized” goats. Mortality due to JD was significantly (*P* < .05) lower in vaccinated groups than in “sham-immunized”. Morbidity rates (due to diarrhea and weakness) were lower in “Bison” group as compared to other groups. Died goats from vaccinated groups showed regression of gross JD lesions and regeneration of fat layer around visceral organs while “Sham-immunized” goats exhibited frank lesions. Vaccinated goats had higher protective CMI response and also higher antibody titer for the trial period as compared to “Sham immunized”. Both vaccines also reduced shedding of MAP in feces significantly (*P* < .05). Though the two vaccines effectively restricted the severity of clinical symptoms of JD, however “Indigenous vaccine” was superior in many respects.

## 1. Introduction

*Mycobacterium avium* subspecies *paratuberculosis* (MAP) is the cause of chronic incurable infection of animals called Johne's disease (JD) and is also associated with Crohn's disease (CD) in human beings [1]. MAP has wide host range and interspecies transmission has been reported frequently [2]. JD causes huge economic losses by way of reduced productivity in domestic ruminants, all over the world. It is estimated that 68.0% of US dairy herds are infected with JD, costing $200 million to $1.5 billion per year to dairy industry [3]. These losses have been neither estimated nor realized in most of the developing and poor countries. In animals vaccination is the only cost effective method for controlling JD. After the first description of a live attenuated vaccine in France [4], many of the developed countries resorted to vaccination and have effectively decreased JD prevalence in their herds and flocks [5–10]. Both killed and live attenuated vaccines have the same efficiency in small ruminants [11]; however, killed vaccine has many advantages in terms of safety, marketing, and storage [12]. Vaccine prepared from field isolate worked better than “commercial vaccine” in calves [13].

Goatherds maintained at Central Institute for Research on Goats (CIRG), Makhdoom were endemic for JD [14–16]. Using “test-and-cull” policy for past 25 years, prevalence of JD could not be reduced. Contrarily the incidence and pathogenicity of MAP increased over the years [14, 15]. Native “S 5” strain of MAP [17] recovered from a terminally sick goat has been characterized as “Indian Bison type” [18] and is most prevalent genotype in India [19]. This native “S 5” strain is highly pathogenic and is a newly evolved genotype as compared to MAP K10 strain [20, 21], therefore, used for the development of ELISA kit [16] and “Indigenous vaccine” for JD [22]. This pilot study was the first randomized trial to know the efficacy of “Indigenous vaccine” in comparison to a “commercial vaccine” for the control of Johne's disease in clinically infected farm goatherds.

## 2. Materials and Methods

### 2.1. Animal Ethics

Institute (CIRG) is registered with Committee for the Purpose of Control and Supervision of Experimentation on Animals (CPCSEA), Government of India, and use of JD-infected goats was approved by the Institute Ethics Committee.

### 2.2. Animals and Management Conditions

Seventy-one discarded ready to cull (stunted/weak/diarrheic) and naturally infected goats with clinical JD (positive in culture and ELISA) from the farm herds of CIRG were randomly divided into 3 groups: “Bison”, “Gudair” and “Sham immunized”, consisting of 29, 24, and 18 goats, respectively. This pilot trial to study the efficacy of “Indigenous vaccine” was truly random, with respect to age, sex, and breed, stage of disease, and body weights of goats. In each group, age of goats varied from 1 to 8.2 years (85.0% were between 2–4 years). Goats in 3 groups were maintained together under semiintensive system of management with 150 gr of concentrate per goat, irrespective of sex, age, body weight, and physiological status. Physically goats were weak and emaciated when transferred from different goats units at CIRG, and JD, being endemic in goats, was primarily responsible for poor health condition of goats [14, 15]. Skin was rough, hard, and dry, eyes were dull and depressed, body weights were below normal, and JD was major cause of morbidity, mortality, and culling of goats.

### 2.3. Vaccines

Two inactivated vaccines: “indigenous vaccine” [22] developed using novel, native, pathogenic, and genetically characterized “S 5” strain of MAP (Indian Bison type) of goat origin [20] and a “Commercial vaccine” (Gudair) manufactured by CZ Veterinaria, Spain [23] were compared for their therapeutic attributes (immune response and improvement in the health status) of naturally infected goats with clinical JD for a period of 210 days. “Indigenous vaccine” [22] contained 2.5 mg (dried weight) of heat inactivated native strain of MAP [20] with 0.01% of Thiomersal suspended in Aluminum hydrooxide gel (CZ Veterinaria, Spain). Dry weight of 2.5 mg contained approximately 12 × 10^8^ bacilli per mL (McFarland standard). “Commercial” vaccine contained 2.5 mg of MAP bacilli (strain 316F) per mL of vaccine in mineral oil.

### 2.4. Vaccination

Goats in “Bison” group were vaccinated with 1 mL of “indigenous vaccine” subcutaneously (behind the ear). Similarly “Gudair” group was vaccinated with 1 mL of “Commercial vaccine” and “Sham-immunized” group was given 1 mL of sterilized PBS.

### 2.5. Data Recordings and Collection of Samples

Body weights of the goats were recorded, zero day post vaccination (DPV) and at 30 DPV intervals up to 210 days. Average gain in body weights by goats of three groups was analyzed using ANOVA. Serum samples of all the goats and blood samples of 9 goats **(**3 from each group) were screened by ELISA and Lymphocyte Transformation Test (LTT), respectively. Nitric oxide (NO) estimation was performed on serum samples of all the goats at 30-day interval. Improvements in body condition, mortality, and morbidity were recorded. Goats that died during the trial period were subjected to detailed necropsy and lesions were recorded. Goats falling sick were treated symptomatically.

### 2.6. Peripheral Blood Mononuclear Cells (PBMCs)

PBMCs were isolated as per method of Boyum [24], using Histopaque (Sigma-Aldrich).

### 2.7. Lymphocyte Transformation Test (LTT)

LTT was performed as per Uma et al. [25], with modifications, on the representative goats (3 each) of the 3 groups (2 vaccinated and 1 Sham immunized) using PBMCs at 60 DPV. Briefly, PBMCs isolated from goats of each group were stimulated in triplicate with mitogen Con A (Sigma) and sonicated protoplasmic antigen of MAP “Indian Bison Type” each at 20 *μ*g/mL concentration, and a set of PBMCs of the same goat was kept as unstimulated control. PBMCs were cultured in 96-well tissue culture plates at 37°C at 5% CO_2_ for 120 hours (5 days). At the end of 120 hours MTT assay was performed and 25 *μ*l of MTT [3-(4, 5- dimethyl trizol-2-yl)-2, 5-diphenyl tetrazolium bromide] dye (5 mg/mL) was added to each well of the tissue culture plate. Plate was incubated at 37°C for 4 hours. After incubation 150 *μ*l of DMSO (dimethyl sulfoxide) was added to each well and incubated overnight at 37°C and absorbance was read at 570 nm. Proliferation of lymphocytes was indicated by optical density value of the well with test samples and that of the unpulsed wells. For analysis of data, a signal-to-noise ratio, that is, Stimulative Index (SI) was calculated for individual goats using the following formula: *Average OD at 570 nm in stimulated wells/Average OD at 570 nm in nonstimulated control wells*. Average SI value for each group of goats was calculated and compared to assess cellular immune response in vaccinated and sham-immunized groups at 60 DPV.

### 2.8. Enzyme Linked Immunosorbent Assay (ELISA)

Pre- and post-vaccination antibody response was measured by ELISA. Antibody titers were monitored in all the goats from zero to 210 DPV at 30-day interval as per Singh et al. [16]. OD values of serum samples were transformed to S/P ratio as described by Collins [26] and goats in strong positive category were considered as positive.

### 2.9. Nitric Oxide (NO) Estimation

Nitric Oxide was estimated at 0, 15, 45, 90 and each 30 DPV after 90 days up to 210 DPV in the serum samples as per Sastry et al. [27]. Optical density (OD) values were transformed into OD index values by division of the mean OD for each serum by the mean OD for the positive control.

### 2.10. Fecal Culture

Fecal culture was performed on zero and 210 DPV from all the goats to check the efficiency of two vaccines in decreasing number of MAP shedders. Fecal samples were grounded, centrifuged, as well as decontaminated (0.9% HPC), and sediment was inoculated on Herrold's egg yolk medium with mycobactin J as per Merkal [28].

## 3. Results

### 3.1. Body Conditions

There was marked improvement in the overall body conditions of vaccinated goats as compared to sham immunized. Vaccinated and Sham-immunized goats could be easily differentiated from distance on the basis of differences in the body conditions. Vaccinated goats were alert, active, healthy, regained skin luster, shining, pliability, regeneration of hairs and had shining bright eyes.

#### 3.1.1. Body Weights

Average body weights, at the time of vaccination in 3 groups, were almost similar (Table 1). At the 210 DPV there was significant difference in the average body weights gained by the goats of vaccinated groups than in sham-immunized group. Goats in “Bison” group gained higher body weights as compared to “Gudair” (*P* > .05) and “sham-immunized” (*P* < .05) groups. Few goats from each group lost weight (in comparison to weight at zero DPV) during the trial period. Percentage of goats loosing body weight was higher in sham-immunized (38.9%) followed by Gudair (33.3%) and Bison (17.2%) groups. Lowered number of vaccinated goats that lost weight after vaccination was indicative of the maximum “individual effect” of the “Indigenous vaccine”.

#### 3.1.2. Mortality Rates and Causes of Deaths

Overall mortality rate was lower in vaccinated groups than in “Sham-immunized” group (Table 2). Percent of goats that died due to JD were 71.4%, 14.2%, and 16.6% in “sham-immunized”, “Gudair”, and “Bison” groups, respectively. On comparing the mortality due to JD between zero and 210 DPV, there were significantly less (*X* ^2^ = 6.28, df = 1, *P* < .02) deaths in the vaccinated groups than in sham-immunized goats.

#### 3.1.3. Morbidity Rate

There was minor outbreak of contagious ecthyma (Orf) in 12 vaccinated goats just after vaccination and lasted for 7 days. In “Bison” group, one goat of Marwari breed suffered from maggotic wound and was under treatment for 22 days. There was significant decrease in the body weight of this goat. In “sham-immunized” group besides diarrhea, pneumonia was frequent cause of sickness. Number of goats sick (due to diarrhea and weakness) and those that received treatment during trial period were higher in sham-immunized group as compared to vaccinated groups.

#### 3.1.4. Gross Lesions of JD at Necropsy

**Figure 1:**
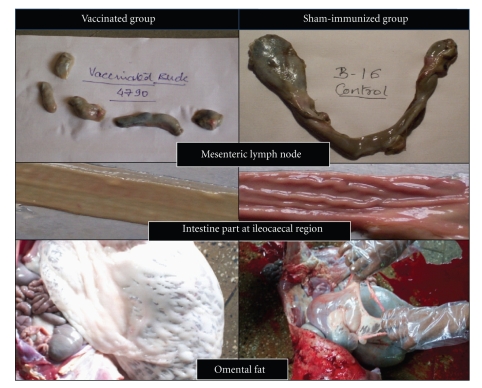
Pictorial presentation for comparative gross lesions (in mesenteric lymph nodes and intestines) and presence of omental fat in vaccinated and sham-immunized goats.

Four goats, out of 7 died in “Sham-immunized group”, had marked gross lesions of JD, and typical acid fast bacilli indistinguishable from MAP were demonstrated in impression smears. Whereas, 2 goats that died at 60 DPV in “Gudair” group showed mildre inflammatory lesions in mesenteric lymph nodes (reduced thickening and size, lowered level of inflammation) and intestines (lowered thickening and no corrugations). Substantial fat layer was generation around all the visceral organs as compared to goats that died in sham-immunized group. Similarly, in “Bison” group also gross lesions of JD were mild in goats that died within few months of vaccination. Marked improvements were seen in the internal condition of one goat of “Bison” group that died at 165 DPV. Wherein gross lesions for JD were very mild, carcass yield was average and there was extensive regeneration of fat layer (visceral fat, kidney fat, cod fat, fat on genital organs, etc.) around all the visceral organs in comparison to sham-immunized goats (Figure 1). This goat died of acidosis.

### 3.2. Cellular Immune Response (CMI) 

#### 3.2.1. Lymphocytes Transformation Test (LTT)

**Figure 2:**
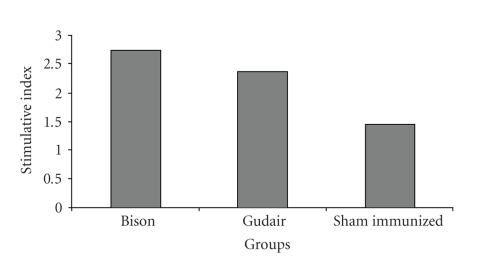
Comparison of Cellular Immune response (Stimulation Index) in two vaccine groups and a sham-immunized group at 60 days post vaccination.

Ability of the PBMCs to recognize and respond to MAP antigen was investigated. PBMCs of vaccinated groups had greater, “Stimulative Index” (SI), when pulsed with protoplasmic antigen from native MAP strain than “Sham-immunized” group at 60 DPV (Figure 2). However, SI was higher in “Bison” as compared to “Gudair” group. Proliferation of cells in response to mitogen was higher as compared to stimulation with MAP in all the three groups.

#### 3.2.2. Nitric Oxide Estimation

**Figure 3:**
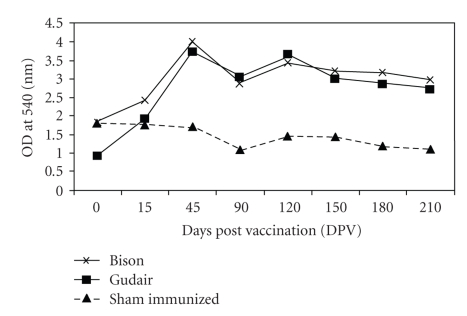
Comparison of Nitric Oxide concentration (Cellular Immune response) in vaccinated and sham-immunized groups at different time intervals.

Results of nitric oxide (NO) production were expressed as geometric means at each sampling interval and are graphically presented in Figure 3. At 0 DPV, concentration of NO in serum was comparable among the 3 groups, but afterwards vaccinated group of goats had higher NO concentration than “sham immunized” (Figure 3). At 45 DPV and onward sampling intervals, both of the vaccinated groups had significantly higher (*P* < .05) concentration of NO as compared to “sham-immunized” group. In “Bison” and “Gudair” groups, peak NO concentration was seen at 45 DPV and subsequently concentration declined slightly at 90 DPV. After 90 DPV at each sampling interval, goats in vaccinated groups maintained NO concentration with slight up and down and remained higher than sham-immunized goats at all the postvaccination intervals.

### 3.3. Humoral Immune Response (ELISA)

**Figure 4:**
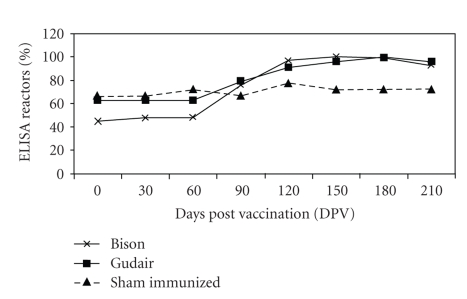
Percent ELISA reactors at different time intervals (days post vaccination).

After vaccination high seroconversion rates were seen in vaccinated goats as compared to “Sham-immunized” (Figure 4). Number of goats were positive in ELISA test at the time of vaccination (0 DPV), since the trial was conducted on discarded goats with advanced stage of MAP infection. However, percent goats seroconverted remained higher in vaccinated groups than in “Sham-immunized” group at all the postvaccination sampling intervals. Almost all the goats in vaccinated groups became seropositive (seroconverted) at 150 DPV (Figure 3).

### 3.4. Fecal Culture

Fecal culture of goats in 3 groups was performed at zero and 210 DPV. Percent of goats positive for MAP at 0 day were 88.8, 79.3, and 79.1 in “sham-immunized”, “Bison”, and “Gudair” groups, respectively. At 210 DPV there was significant reduction in shedding of MAP (Bison: *X* ^2^ = 4.293, *P* = .038; Gudair: *X* ^2^ = 3.938, *P* = .047) in feces of goats in “Bison” and “Gudair” groups. Two vaccines reduced shedding of MAP in feces dramatically and only 17.2 and 29.1% goats were excreting MAP in “Bison” and “Gudair” groups, respectively, at 210 DPV. In “sham-immunized” group, all the goats (18 = 100%) were positive for excretion of MAP at 210 DPV.

## 4. Discussion

Vaccination has been known to offer good protection and recovery against MAP infection and shown to reduce the prevalence of clinical JD by 90% and prevalence of JD in herds by about 50% [29]. First Indian “Indigenous vaccine” against JD in goats [22] was developed using most prevalent and highly pathogenic “S 5” strain of *Mycobacterium avium* subspecies *paratuberculosis* (MAP) genotyped as “Indian Bison Type”. This “indigenous vaccine” has been shown to provide good protection (prophylactic) in experimentally vaccinated and twice-challenged goats [22]. There are a number of reports on the prophylactic properties of JD vaccine, whereas very few reports are available on the therapeutic potential. Substantial reports exist on the significant reduction in mortality, clinical symptoms, and excretion of MAP bacilli in feces after vaccination of infected goats, thereby reducing environmental contamination and providing less opportunity for disease transmission.

Sigurdsson and Gunnarson [10] achieved successful eradication of JD in Iceland by vaccinating lambs once with killed vaccine. Similar results were reported by Crowther et al. [6] in sheep in Cyprus by vaccinating all stocks with live attenuated vaccine. Similarly in Norway, after several years of unsuccessful efforts to eradicate JD in goats (isolation and slaughter), vaccination program with “live attenuated vaccine” for kids was started in 1967 and the infection of MAP was reduced from 53% to 1% [30]. In 1964, Great Britain introduced a scheme for the voluntary vaccination of the stock on infected farms and freedom from clinical disease was achieved after an average of 4 years [31]. In 1983, a control program based on killed vaccine was initiated in The Netherlands and this strategy has been successful in reducing clinical JD and was less expensive than subsidized cull and slaughter program [7].

Results of the present study also indicated that vaccination could be practiced in any stage of disease (sub-clinical, clinical, and advance clinical), in any age group (kids above 3 months old to adults) and in any physiological stage (dry, lactating, or pregnant). Initial priming of the goats with MAP infection at the start of trial could have elicited higher immune response against vaccine (second inoculation of the antigen). JD was endemic in CIRG herds; therefore, continuous and slow reinfection of vaccinated animals from environment might have booster effect, thereby complete turnaround in the condition of ready to cull goats when trial was started. Incomplete degree of the response in 15-day-old kids as observed by Corpa et al. [32] might be due to immature immune system at that age. JD vaccination has been practiced during first weeks of life on the basis that the protection would be conferred for the first contact with mycobacteria [30, 33]. However, other studies on vaccination of adult animals also showed very good results in controlling JD [6, 9]. Corpa et al. [32] also showed that immune response was higher in adult animals as compared to few weeks old animals.

Lymphoproliferative response to antigen stimulation has been widely used as in vitro correlates of cell-mediated immunity [34]. PBMCs from vaccinated and “sham-immunized” goats were used to assess the response to protoplasmic antigen of MAP. Proliferative response was higher in PBMCs from vaccinated groups on stimulation with MAP antigen than in PBMCs from “sham-immunized” group. However, stimulative index was higher in PBMCs from “Bison” than in “Gudair” group. Low responsiveness may be attributed to the suppressive factors secreted by monocytes and lymphocytes [35]. Another possibility may be a shift from Th l to Th 2 type of cytokine response [36]. Further preferential sequestration of antigen-specific T cells into the infected areas leads to their absence in peripheral blood [37].

It is generally believed that reactive nitrogens, such as NO, are most effective in direct killing of mycobacteria [38]. Significantly higher NO concentration was seen in vaccinated groups (maximum in “Bison” group and least for “sham-immunized” group). Increased production of NO induced by antigen may cause effective immune response towards MAP and may lead to inhibition of MAP in macrophages. 

Significant rise in the peripheral blood antibodies was seen in vaccinated groups after vaccination and “peak titers” were attained at 60 DPV that gradually declined, such pattern was absent in “sham-immunized” group. Comparative antibody titers, in the vaccinated groups, were higher in “Bison” group. In vaccination of sheep by a killed vaccine maximum ELISA reactors were observed around 30 DPV [39]. In a study, 13 of the 15 vaccinated calves also became ELISA positive within 60 to 360 DPV [40]. In some goats of vaccinated group there was again rise in the antibody titer at 180 DPV. But this rise in the antibody titer was less than the primary maxima observed at 60 DPV. Although antibody response may not have the protective effect, it would be an indicator of the degree of the activation of immune system against mycobacteria [32]. Th1 and Th2 responses are not antagonist and IFN*γ* can play stimulatory effect on B-lymphocytes and antibody production [41]. Antibody titers were higher in few goats of “sham-immunized” group but titers were low as compared to vaccinated groups.

There was decreasing trend of NO concentration in “sham-immunized” goats. Probably protective Th1 response decreased due to decrease in the concentration of pro-inflammatory cytokines (IFN*γ*, IL-12, IL-2, TNF*α*, etc.). Though NO concentration also showed decreasing trend in vaccinated goats, they still maintained significantly higher concentration of NO as compared to “sham-immunized” goats. These results were further supported by antibody results, in “sham-immunized” goats, where titers increased slightly towards the end of the experiment. As “‘sham-immunized” goats approached terminal stage of JD, inhibitory Th2 responses might be increased with suppressed activity of antiinflammatory cytokines to limit the host tissue damage.

Necropsy of goats from “sham-immunized” group exhibited marked gross lesions of JD. Lymph nodes were highly enlarged and swollen, intestinal mucosa was thickened with prominent ridges (corrugation), and degeneration of visceral fat was marked in these goats. Goats from vaccinated groups did not show severe lesion of JD grossly due to remission of lesions after vaccination. In a study of vaccination in infected animals, Corpa et al. [32] also reported reduction in progression of granulomatous lesions and in bacterial shedding. Similarly, Juste et al. [42] also noticed regressive type granulomas (tuberculoid forms of lesions) located exclusively in the intestinal organized lymphoid tissues in vaccinated animals, whereas in nonvaccinated animals lesions spread to other areas of intestine causing severe enteritis. Therefore, vaccination may have potential to direct the inflammatory cascade into a beneficial mode.

Vaccination reduced fecal shedding in significant number of vaccinated goats. Initially, 16 (88.8%) goats were positive in “sham-immunized” group, but at 210 DPV all the goats were positive in fecal culture. At zero and 210 DPV, the number of goats excreting MAP decreased from 23 (79.3%) and 19 (79.1%) to 5 (17.2%) and 7 (29.1%) in “Bison” and “Gudair” groups, respectively. Number of goats negative for MAP infection were more in “Bison” group. Colony-forming units (cfu) counts also decreased in vaccinated goats as compared to 0 DPV. However, in “sham-immunized” group, cfu increased in 55.0% of goats.

Vaccination of lambs against ovine JD reduced deaths up to 90% and also reduced amount of bacilli passed in feces by up to 90% [8]. Rapid decrease in new clinical cases was achieved following immunization of infected adult sheep flocks having severe clinical disease [9]. Similar results have been reported by Uzonna et al. [13], where vaccine prepared from field isolate reduced the number of fecal shedders more than that of commercial vaccine.

Present trial indicated that “Indigenous vaccine” used in this study exhibited “Therapeutic effect” by reversing the clinical signs. There was reduction in shedding of number of MAP bacilli per goat, number of goats with clinical, JD and number of goats positive bacteriologically (fecal shedders), and complete turnaround was visible in the physical condition of the vaccinated goats as compared to “sham immunized”. Internally the lesions of JD reduced and there was regeneration of visceral fat layer and fat layer around all the visceral organs. Goat became alert active, and udders were filled with milk despite continuous suckling by kids. “Indigenous vaccine” using aluminum hydrooxide gel as adjuvant was superior to “Gudair” using mineral oil, in controlling JD in the naturally infected goats. However, these minor differences in vaccine-induced immune response may have resulted due to use of two different adjuvants, differences in the genetic makeup of individual animals and level of MAP infection.

## 5. Conclusions

Single dose of “Indigenous vaccine” developed from highly pathogenic locally isolated “Indian Bison Type” genotype of MAP significantly reduced morbidity and mortality, reversed clinical signs as well as reduced shedding of MAP, and there was marked improvement in physical and internal body condition (Therapeutic effect). Therefore, “Indigenous vaccine” worked as “Therapeutic Vaccine” in goats suffering from clinical to advance clinical symptoms of JD.

## Figures and Tables

**Table 1:** Average body weights gained per goat, by vaccinated and “Sham-immunized” groups.

Groups	Average body weights (kg)	Average body weights gained/goat (kg)
	± SE at 0 DPV*	± SE (0 DPV–210 DPV)
Bison	23.65 ± 0.67	5.49 ± 1.04
Gudair	24.10 ± 0.54	2.75 ± 0.89
Sham immunized	23.05 ± 0.50	1.23 ± 1.15

*DPV: Days post vaccination.

**Table 2:** Mortality rates and causes of deaths during vaccination trial.

Causes of deaths	“Sham immunized”	“Bison”	“Gudair”
Other than JD*	2	6	5
	(28.6)	(85.7)	(83.3)
Johne's Disease	5	1	1
	(71.4)	(14.2)	(16.6)
Goats died/total (Mortality rates)	5	1	1
	(38.9)	(24.1)	(25.0)

Causes of deaths other than JD*: Pneumonia/*Haemonchous/*weakness/

Acidosis; Figures in parentheses are percent.

## Abbreviations

MAP: *Mycobacterium avium *subspecies* paratuberculosis *
JD: Johne's disease
LTT: Lymphocyte transformation test
NO: Nitric Oxide
MTT: 3-(4, 5-dimethyl trizol-2-yl)-2, 5-diphenyl tetrazolium bromide
DMSO: dimethyl sulfoxide
CMI response: Cell-mediated immune response
ELISA: Enzyme-linked immunosorbent assay
cfu: Colony-forming unit
SI: Stimulative index
OD: Optical density,
DPV: Days post vaccination
gr: Gram.

## References

[1] Chiodini RJ, Van Kruiningen HJ, Merkal RS, Thayer WR, Coutu JA (1984). Characteristics of an unclassified *Mycobacterium* species isolated from patients with Crohn’s disease. *Journal of Clinical Microbiology*.

[2] Ayele WY, Machácková M, Pavlík I (2001). The transmission and impact of paratuberculosis infection in domestic and wild ruminants. *Veterinarni Medicina*.

[3] Ott SL, Wells SJ, Wagner BA (1999). Herd-level economic losses associated with Johne’s disease on US dairy operations. *Preventive Veterinary Medicine*.

[4] Valleé H, Rinjard P, Valleé M (1934). Sur la prémunition de l’enterite paratuberculeuse des bovides. *Revue Générale De Médecine Vétérinaire*.

[5] Benedictus G (1984). Evaluation of the organized control of paratuberculosis in the province of Friesland. *Tijdschrift voor Diergeneeskunde*.

[6] Crowther RW, Polydorou K, Nitti S, Phyrilla A (1976). Johne’s disease in sheep in Cyprus. *Veterinary Record*.

[7] Dijkhuizen AA, Schaik GV, Huirne RMB, Kalis CHJ, Benedictus G (1994). Cost benefit analysis of vaccination against paratuberculosis in dairy cattle. *Kenya Veterinarian*.

[8] Meat and livestock Australia (MLA).

[9] Perez V, Garcia Marin JF, Bru R, Moreno B, Badiola JJ (1995). Results of vaccination of adult animals against ovine paratuberculosis. *Medicina Veterinaria*.

[10] Sigurdsson S, Gunnarson E Paratuberculosis in sheep, goats and reindeer in Iceland: a result of an import of a flock of sheep from Germany 1933. The control of disease.

[11] Garcia Marin JF, Tellechea J, Gutierrez M, Corpa JM, Perez V Evaluation of two vaccines (killed and attenuated) against small ruminant paratuberculosis.

[12] Huitema H (1967). Johne’s disease in cattle and vaccination. *Bulletin de l’Office International des Epizooties*.

[13] Uzonna JE, Chilton P, Whitlock RH, Habecker PL, Scott P, Sweeney RW (2003). Efficacy of commercial and field-strain *Mycobacterium paratuberculosis* vaccinations with recombinant IL-12 in a bovine experimental infection model. *Vaccine*.

[14] Kumar P, Singh SV, Bhatiya AK (2007). Juvenile Capri-Paratuberculosis (JCP) in India: incidence and characterization by six diagnostic tests. *Small Ruminant Research*.

[15] Kumar S, Singh SV, Sevilla I (2008). Lacto-incidence and evaluation of 3 tests for the diagnosis of Johne’s disease using milk of naturally infected goatherds and genotyping of *Mycobacterium avium* subspecies *paratuberculosis*. *Small Ruminant Research*.

[16] Singh SV, Singh AV, Singh PK, Gupta VK, Kumar S, Vohra J (2007). Sero-prevalence of paratuberculosis in young kids using ‘Bison type’, *Mycobacterium avium* subsp. *paratuberculosis* antigen in plate ELISA. *Small Ruminant Research*.

[17] Hajra S, Singh SV, Srivastav AK, Neilsen SS Pathobiology of spontaneous and experimental paratuberculosis (S-5 strain) in goats with special reference to early lesions.

[18] Sevilla I, Singh SV, Garrido JM (2005). Molecular typing of Paratuberculosis strains from different hosts and regions. *OIE Revue Scientifique et Technique*.

[19] Singh SV, Sohal JS, Singh PK, Singh AV (2009). Genotype profiles of Mycobacterium avium subspecies paratuberculosis isolates recovered from animals, commercial milk, and human beings in North India. *International Journal of Infectious Diseases*.

[20] Sohal JS, Sheoran N, Narayanasamy K, Brahmachari V, Singh SV, Subodh S (2009). Genomic analysis of local isolate of *Mycobacterium avium* subspecies *paratuberculosis*. *Veterinary Microbiology*.

[21] Sohal JS, Singh SV, Singh PK, Singh AV (2010). On the evolution of ‘Indian Bison type’ strains of *Mycobacterium avium* subspecies *paratuberculosis*. *Microbiological Research*.

[22] Singh SV, Singh PK, Singh AV, Sohal JS, Gupta VK (2007). Comparative efficacy of an indigenous ‘inactivated vaccine’ using highly pathogenic field strain of *Mycobacterium avium* subspecies *paratuberculosis* ‘Bison type’ with a commercial vaccine for the control of Capri-paratuberculosis in India. *Vaccine*.

[23] Britton A Safety and efficacy assessments of killed mycobacterial vaccine-Gudair^TM^ for use in the control of ovine Johne’s disease.

[24] Böyum A (1968). Separation of leukocytes from blood and bone marrow. *Scandinavian Journal of Clinical and Laboratory Investigation*.

[25] Uma H, Selvaraj P, Reetha AM, Xavier T, Prabhakar R, Narayanan PR (1999). Antibody and lymphocyte responses to mycobacterium tuberculosis culture filtrate antigens in active and, quiescent (cured) pulmonary tuberculosis. *Indian Journal of Tuberculosis*.

[26] Collins MT (2002). Interpretation of a commercial bovine paratuberculosis enzyme-linked immunosorbent assay by using likelihood ratios. *Clinical and Diagnostic Laboratory Immunology*.

[27] Sastry KVH, Moudgal RP, Mohan J, Tyagi JS, Rao GS (2002). Spectrophotometric determination of serum nitrite and nitrate by copper-cadmium alloy. *Analytical Biochemistry*.

[28] Merkal RS (1984). Paratuberculosis: advances in cultural, serologic, and vaccination methods. *Journal of the American Veterinary Medical Association*.

[29] Lambert G Paratuberculosis: prevalence, diagnosis, prevention and treatment.

[30] Saxegaard F, Fodstad FH (1985). Control of paratuberculosis (Johne’s disease) in goats by vaccination. *Veterinary Record*.

[31] Wilesmith JW (1982). Johne’s disease: a retrospective study of vaccinated herds in Great Britain. *British Veterinary Journal*.

[32] Corpa JM, Peérez V, García Marín JF (2000). Differences in the immune responses in lambs and kids vaccinated against paratuberculosis, according to the age of vaccination. *Veterinary Microbiology*.

[33] Larsen AB, Hawkins WW, Merkal RS (1964). Experimental vaccination of sheep against Johne’s disease. *American Journal of Veterinary Research*.

[34] Pitchappan RM, Brahmajothi V, Rajaram K, Subramanyam PT, Balakrishnan K, Muthuveeralakshmi R (1991). Spectrum of immune reactivity to mycobacterial (BCG) antigens in healthy hospital contacts in South India. *Tubercle*.

[35] Kleinhenz ME, Ellner JJ (1987). Antigen responsiveness during tuberculosis: regulatory interactions of T cell subpopulations and adherent cells. *Journal of Laboratory and Clinical Medicine*.

[36] Surcel HM, Blomberg MT, Paulie S (1994). Th1/Th2 profiles in tuberculosis, based on the proliferation and cytokine response of blood lymphocytes to mycobacterial antigens. *Immunology*.

[37] Rossi GA, Balbi B, Manca F (1987). Tuberculous pleural effusions. Evidence for selective presence of PPD-specific T-lymphocytes at site of inflammation in the early phase of the infection. *American Review of Respiratory Disease*.

[38] Mullerad J, Hovav AH, Nahary R, Fishman Y, Bercovier H (2003). Immunogenicity of a 16.7 kDa *Mycobacterium paratuberculosis* antigen. *Microbial Pathogenesis*.

[39] Eppleston J, Britton A, Windsor P, Hall D, Whittington R, Jones S Progress in a field trial to determine the effectiveness of a killed *Mycobacterium paratuberculosis* vaccine for the control of OJD in Australian sheep flock.

[40] Spangler E, Heider LE, Bech-Nielsen S, Dorn CR (1991). Serologic enzyme-linked immunosorbent assay responses of calves vaccinated with a killed *Mycobacterium paratuberculosis* vaccine. *American Journal of Veterinary Research*.

[41] Abbas AK, Murphy KM, Sher A (1996). Functional diversity of helper T lymphocytes. *Nature*.

[42] Juste RA, Garcia-Marin JF, Peris B, de Ocariz CS, Badiola JJ (1994). Experimental infection of vaccinated and non-vaccinated lambs with *Mycobacterium paratuberculosis*. *Journal of Comparative Pathology*.

